# Autoimmunity in Reproductive Health and Pregnancy

**DOI:** 10.1155/2018/9501865

**Published:** 2018-02-13

**Authors:** Jacek Tabarkiewicz, Senthamil R. Selvan, Nathalie Cools

**Affiliations:** ^1^Department of Human Immunology, Faculty of Medicine, University of Rzeszów, Rzeszów, Poland; ^2^Centre for Innovative Research in Medical and Natural Sciences, Faculty of Medicine, University of Rzeszów, Rzeszów, Poland; ^3^Omni Array Biotechnology, Rockville, MD 20855, USA; ^4^Immune Regulation & Tolerance-Inducing Strategies (IRiS) Research Group, University of Antwerp, Antwerp, Belgium

The influence of pregnancy on the maternal immune system is complex and orchestrated by multiple hormonal and metabolic factors provided by the mother as well as the fetus. The modifications of the maternal immune response include changes in cell proportions, phenotypes, and their functional ability to produce cytokines and other mediators. During pregnancy, activations of pro- and anti-inflammatory responses are fluently regulated depending on the phase of pregnancy [1]. Implantation, placentation, and delivery phases are proinflammatory, while the period of rapid fetal growth and development is anti-inflammatory [1]. The model of “immune suppression” during pregnancy has been long accepted, yet at the present, we are aware that fetal tolerance is not caused by suppression of the maternal immune system but rather by immunomodulation and that pregnant women are very much capable of having robust immune responses [2]. An understanding of the balance between tolerance and protection of the fetus and maternal active immune response against pathogens or self-antigens may contribute to developing new approaches to the problem of autoimmunity in reproductive health and pregnancy.

Autoimmune diseases are characterized by organ and tissue damage caused by self-reactive immune responses mediated by antibodies and/or T cells. These diseases may be associated with genetic and/or environmental predispositions. Thus, autoimmune disorders predominantly affect women and often occur during reproductive years and have implications for fertility and pregnancy to some extent [3, 4]. The relationships between autoimmunity and reproduction include the impact of pregnancy on the clinical course of autoimmune disorders as well as the influence of autoimmunity on pregnancy development. Thus, in this population of patients, specialized concerns in pregnancy planning and management are commonly encountered. Autoimmune diseases are usually thought to be associated with pathogenic activity of Th17-/Th1-type cells; during pregnancy, Th2-type cytokines are noted to be crucial to maintain the tolerance of the mother towards the fetal semiallograft [3, 4]. In pregnancy, immunoregulatory cytokines and cells are present in the mother's circulatory system and accumulate in the decidua and can modify autoimmune responses influencing the symptoms of autoimmune disease.

Systemic lupus erythematosus (SLE), primary antiphospholipid syndrome (APS), and secondary SLE-associated APS affect mostly women of childbearing age [5]. In a paper by Andreoli et al., we found the most current recommendations of the European League Against Rheumatism (EULAR) for women's health issues in SLE and/or APS that were developed using an evidence-based approach followed by an expert consensus [5]. The considerations in the expert consensus were as follows: family planning, reduction of the risks of adverse maternal or fetal outcomes, use of hormonal contraception and menopause replacement therapy, fertility preservation during therapy, assisted reproduction techniques, importance of disease activity assessment, fetal monitoring, risk of gynecological malignancies associated with exposure to immunosuppressive drugs, and human papillomavirus immunization.

In this special issue, original research articles will focus on the most recent advances on the influence of autoantibodies on fertilization, the impact of SLE, APS, and endometriosis on pregnancy.

The anticentromere antibody (ACA) could impair oocyte maturation potential and early embryonic development, but the mechanisms of this pathology are still unknown. Thus, Y. Ying et al. conducted a preliminary exploration to determine if ACA could penetrate into living mouse embryos and impair their developmental potential. An immunofluorescence assay was performed to determine penetration of polyclonal anticentromere antibodies in the embryos. They show that anti-CENP-A antibody binds to the nucleus of exposed embryos which results in significant growth impairment. These novel results based on *in vitro* observations provide a direction for studies considering mechanisms of ACA influence on embryos and confirmation of these findings in *in vivo* studies.

Antinuclear antibodies (ANAs), including anti-dsDNA antibodies, could be associated with infertility, a decline of oocyte quality and impairment of embryo development, recurrent spontaneous abortion, and *in vitro* fertilization (IVF) failure. Research by J. Fan and colleagues explored whether the anti-dsDNA antibody could adversely affect reproductive outcomes. A total of 259 women receiving *in vitro* fertilization-embryo transfer cycle (IVF) were enrolled in this study including women positive for ANA and anti-dsDNA, positive for ANA and negative anti-dsDNA, and negative for ANA and anti-dsDNA. They compared the number of retrieved oocytes, available embryos and high-quality embryos, rates of implantation, clinical pregnancy and abortion among three groups in fresh embryo transfer, and frozen-thawed embryo transfer cycles, respectively. The authors found that all pregnant patients suffered from abortion in the ANA+/anti-dsDNA+ group which suggests the need of new approaches to understand the role of autoantibodies, especially the involvement of anti-dsDNA, in reproductive outcomes.

Systemic lupus erythematosus is a chronic, multisystem autoimmune disease mostly affecting females of childbearing age and provides challenges in the prepregnancy, antenatal, intrapartum, and postpartum periods for patients and the medics providing care. S. J. Kroese and coauthors had the opportunity to pool data from the rheumatology and obstetric departments of two tertiary centers during a 16-year period. They investigated SLE disease activity before, during, and after pregnancy. Moreover, they examined maternal and perinatal complications in this population, antiphospholipid antibody status, pregnancy complications, and the total number of live births from SLE patients. The authors concluded that in the examined SLE population, the incidence of maternal and perinatal complications is high compared to the reported rates in the general population, irrespective of antiphospholipid antibody status, and despite low disease activity before, during, and after pregnancy. These results obtained during long period observational study could be of additional value in the counseling of SLE patients.

Therapy of rheumatic disorders with antimalarials, especially hydroxychloroquine (HCQ), is well confirmed. The research paper authored by S. J. Kroese et al. investigates if HCQ treatment during pregnancy in women with SLE is associated with improved pregnancy outcomes. The analyzed data was obtained at the University Medical Center Utrecht between 2000 and 2015, and sixty-three SLE patients were included. Hydroxychloroquine use was associated with longer pregnancy duration in the vulnerable preterm birth population, underscoring a beneficial effect of HCQ use during pregnancy. However, the authors state that retrospective and observational character of their study could be its limitation.

Antiphospholipid syndrome is a leading acquired cause of thrombosis and pregnancy loss. High mobility group box 1 (HMGB1) is nuclear protein which organizes DNA and regulates transcription. Furthermore, HMGB1 could play a pivotal role in chronic inflammation and autoimmune diseases. In the manuscript “Elevated Serum Level of HMGB1 in Patients with the Antiphospholipid Syndrome” by V. Manganelli et al., the authors described that HMGB1/sRAGE may play a pathogenic role in recurrent abortion in primary and secondary APS. They analyzed sera from 30 patients with antiphospholipid syndrome (11 primary and 19 secondary APS), 35 subjects with pregnancy morbidity, and 30 healthy females for HMGB1 and its putative receptor RAGE (sRAGE) as well as for TNF. The authors suggest that monitoring HMGB1/sRAGE together with other prognostic parameters represents a useful tool to evaluate risk stratification in prevention of adverse pregnancy outcomes and further studies are warranted.

Endometriosis (EMS) is a common gynecologic disease which could be associated with infertility in women. The role of menstruation back flow planting and defects in the immune system in the pathogenesis of EMS are well established and widely accepted. Currently, we know that endometriosis shares similarities with autoimmune disorders, which include elevated levels of cytokines, decreased apoptosis, autophagy, and cell-mediated pathologies. In their original research paper, M. Gogacz et al. demonstrate that in patients with EMS, peritoneal fluid (PF) macrophages express CD95+ at a significantly higher level than in a nonendometriotic group. Moreover, the concentration of the soluble form of Fas in PF of patients with moderate and severe endometriosis was significantly higher when compared to controls. The authors suggest that in the peritoneal cavity of patients with EMS, a local immune response could not be effective in elimination of misplaced endometrial cells because of increased apoptosis of immune cells like macrophages.

In summary, this special issue covers many important aspects of bidirectional associations between autoimmunity and reproductive health and pregnancy. We hope that this special issue can provide valuable information to researchers as well as clinicians and lead not only to enhancement of knowledge but also to serve for developing better care of pregnant patients with autoimmune diseases.
